# Efficacy of non-invasive diagnostic methods in the diagnosis and screening of oral cancer and precancer

**DOI:** 10.1016/j.bjorl.2020.12.019

**Published:** 2021-02-13

**Authors:** Do Hyun Kim, Sun Won Kim, Se Hwan Hwang

**Affiliations:** a The Catholic University of Korea, College of Medicine, Seoul St. Mary’s Hospital, Department of Otolaryngology-Head and Neck Surgery, Seoul, Republic of Korea; b The Catholic University of Korea, College of Medicine, Bucheon St. Mary’s Hospital, Department of Otolaryngology-Head and Neck Surgery, Seoul, Republic of Korea

**Keywords:** Mouth neoplasms, Chemiluminescence, Tolonium chloride, Autofluorescence, Narrow band imaging

## Abstract

**Introduction:**

Traditional meta-analyses on the diagnostic accuracy of oral lesions have been conducted, but they were inherently limited to direct pairwise comparisons between a single method and a single alternative, while multiple diagnostic options and the ranking thereof were methodologically not possible.

**Objective:**

To evaluate the diagnostic values of various methods in patients with oral potential malignant disease by performing a network meta-analysis.

**Methods:**

Two authors independently searched the databases (MEDLINE, SCOPUS, the Cochrane Register of Controlled Trials, and Google scholar) up to June 2020 for studies comparing the diagnostic accuracy of various tools (autofluorescence, chemiluminescence, cytology, narrow band imaging, and toluidine blue) with visual examination or other tools. The outcomes of interest for this analysis were sensitivity, specificity, negative predictive value, positive predictive value and accuracy. Both a standard pairwise meta-analysis and network meta-analysis were conducted.

**Results:**

Treatment networks consisting of six interventions were defined for the network meta-analysis. The results of traditional meta-analysis showed that, among six methods, narrow band imaging showed higher sensitivity, specificity, negative predictive value, positive predictive value, and accuracy compared to visual examination. The results of network meta-analysis showed that autofluorescence, chemiluminescence, and narrow band imaging had higher sensitivity compared with visual examination, and that chemiluminescence and narrow band imaging had higher negative predictive value compared with visual examination. However, autofluorescence and chemiluminescence had lower specificity compared with visual examination. There were no significant differences in positive predictive value and accuracy among the six interventions.

**Conclusion:**

This study demonstrated that narrow banding imaging has superiority in terms of sensitivity and negative predictive value compared with the other five tested agents.

## Introduction

Despite the remarkable development of surgical management and adjuvant therapies for oral squamous cell carcinoma, the prognosis of patients remains poor with no significant change in the 5-year survival rate for decades.^1^ Therefore, the importance of early detection before cancer progresses has been increasingly highlighted. Oral potential malignant disease (OPMD) has been consistently gaining evidence as a precancerous stage.^2^ Therefore, early detection of OPMDs plays an important role in improving prognosis. However, it relies solely on the clinician’s ability to distinguish these lesions from benign conditions. Because OPMD could be asymptomatic and may assume a benign clinical appearance, it could be difficult to distinguish from reactive or inflammatory conditions of the oral mucosa.^3^ Since histopathological examination of the biopsy specimen is the gold standard for diagnosing oral malignancy, its dependency on the clinical experience of the medical practitioner to differentiate cancerous lesions from benign lesions remains an important issue.^4^ Therefore, various techniques such as vital staining, light-based detection, optical diagnostic techniques, and oral cytology have been used to compensate for low clinician reliability; these techniques are aimed at facilitating the early diagnosis of oral cancer.^3^

Traditional meta-analyses on the adjunctive diagnostic accuracy of oral lesions have been conducted, but they were inherently limited to direct pairwise comparisons between a single method and a single alternative, while multiple diagnostic options and the ranking thereof were methodologically not possible. By contrast, a network meta-analysis (NMA) can be used to compare multiple treatment options simultaneously, as it combines all direct and indirect evidence from related studies. Moreover, NMA provides a ranking of the assessed treatment options, thus allowing clinicians to choose the most effective approach as determined statistically.^5^ In this review, we present the results of our NMA of the efficacy of six different adjunctive diagnostic tools used during oral lesions. The evidence-based data can simplify clinical decision-making for the diagnosis of patients with OPMD or cancerous lesions.

## Methods

### Ethical considerations

This review study did not treat human participants. Therefore, our Institutional Review Board waived the need for informed consent for this systematic review and meta-analysis.

### Search strategy and selection of studies

**Figure 1:**
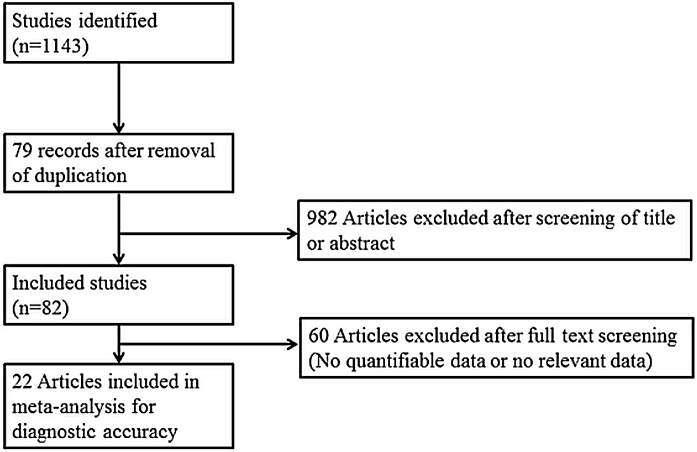
Diagram of the study selection process.

The search strategy was designed and reviewed by a clinical librarian, an information specialist with 10 years of experience. Searching of the database including MEDLINE, Scopus, the Cochrane Register of Controlled Trials, and google scholar was performed in June 2020. We also checked the reference lists of included studies and existing systematic reviews to identify relevant articles. Strategies used are listed in Supplementary Tables 1 to 3, and diagrams of the study selection process are shown in Fig. 1. Overlapping or irrelevant studies were excluded by two independent reviewers screening titles and abstracts, and re-assessment of full texts of potentially eligible articles was also completed. Any discrepancy in the selection of literature was resolved by consensus or the third author.

The inclusion criteria were: 1) use of non-invasive adjunctive diagnostic tools (autofluorescence, chemiluminescence, cytology, narrow band imaging (NBI), and toluidine blue); 2) prospective or retrospective study protocol; 3) comparison of non-invasive adjunctive diagnostic tools with other tools or visual examination; and 4) sensitivity and specificity analyses. The exclusion criteria were: 1) case report format; 2) review article format; 3) diagnosis of other tumors (laryngeal cancer or nasal cavity tumors); and 4) lack of diagnostic data. Missing or incomplete data in the included studies were directly obtained from the authors. This NMA is based on the Preferred Reporting Items of Systematic Reviews and Meta-analyses for NMA.

### Data extraction and risk of bias assessment

Data extraction was performed from included studies using standardized forms by two authors independently. The evaluated outcomes consisted of sensitivity, specificity, negative predictive value, positive predictive value, and accuracy.^2^, ^6^, ^7^, ^8^, ^9^, ^10^, ^11^, ^12^, ^13^, ^14^, ^15^, ^16^, ^17^, ^18^, ^19^, ^20^, ^21^, ^22^, ^23^, ^24^, ^25^, ^26^, ^27^, ^28^, ^29^, ^30^, ^31^, ^32^, ^33^ Accuracy were calculated as (true-positives + true-negatives)/(true-positives + true-negatives + false-negatives + false-positive).^34^ The outcomes were compared with respect to the other diagnostic strategies and the visual examination. From all studies, data were collected regarding the number of patients, the true-positive, true-negative, false-positive, and false-negative values. Study quality was analyzed using the Quality Assessment of Diagnostic Accuracy Studies tool (ver. 2; QUADAS-2).

### Statistical analysis

Meta-analysis was performed using R 3.5.0 ‘netmeta’ package (R Foundation for Statistical Computing, Vienna, Austria). A random-effects NMA within a frequentist framework was conducted^34^ to achieve combined results in the form of odds ratio and 95% Confidence Intervals (95% CIs) for use across all studies to assess sensitivity, specificity, Negative Predictive Value (NPV), Positive Predictive Value (PPV), and accuracy.^35^ In all other cases, the outcomes of the incidence analysis were assessed using odds ratios. To rank treatment options graphically, the surface under the cumulative ranking curve (SUCRA) and mean ranks were adopted. SUCRA represents the probability of a treatment ranking best.^36^ In this study, SUCRA ranged from 0 to 1, with 1 indicating that the treatment option was statistically best and 0 the worst. Direct and indirect comparisons were adequately homogeneous. A loop-specific approach was used to distinguish heterogeneity in all triangular or quadratic loops in the NMA model.^37^ The discrepancy between direct and indirect evidence with the 95% CI was used to distinguish heterogeneity in all loops. Heterogeneity was defined as the difference between direct and indirect evidence with a 95% CI excluding 0. The node-splitting model was used to distinguish heterogeneity between the direct and indirect evidence.^38^ In addition, we made comparison-adjusted funnel plots to assess potential publication bias.^39^

## Results

The literature review yielded 24 studies consisting of 1914 participants. Table 1 is the summary of study characteristics and bias assessment.

**Table 1:** Summary of the studies included in the network meta-analysis.

Study	Year	Nationality	Diagnostic standard of malignant or highly suspicous	Age	Number	Sex	Type	Lesion	Diagnostic modality
Allegra	2009	Italy	Invasive carcinoma or all dysplasia	59 (42–82)	32	19/13	Pros	45	T vs. V
Amirchaghmaghi	2018	Iran	Invasive carcinoma or all dysplasia	52.3 ± 14.8	45	21/24	CS	54	A vs. V
Awan	2015	Pakistan	Invasive carcinoma or all dysplasia	NR	116	65/51	Pros	116	A vs. C vs. T
Chaudhry	2016	India	Invasive carcinoma or all dysplasia	45	100	74/26	Retro	100	C vs. T
Guneri	2011	Turkey	Invasive carcinoma or all dysplasia	NR	35	NR	Pros	43	T vs. A vs. cy
Hanken	2013	Germany	Invasive carcinoma or all dysplasia	41–76	60	25/35	Pros	60	A vs. V
Jayaprakash	2009	USA	Invasive carcinoma or all dysplasia	59.8 (12.5)	60	41/19	Pros	249	V vs A
Kammerer	2015	Germany	Invasive carcinoma or all dysplasia (from moderate)	60.4	44	25/19	Pros	50	T vs. C vs. V
Mehrotra	2010	India	Invasive carcinoma or all dysplasia	41	156	140/16	CS	156	A vs. C
Mojsa	2012	Poland	Invasive carcinoma or all dysplasia	NR	30	NR	Pros	41	T vs. V
Petruzzi	2014	Italy	Invasive carcinoma or all dysplasia	56.7	49	22/27	Pros	56	A vs. T
Piazza	2016	Italy	Dysplasia (mild-moderate), CIS, Cancer	NR	128	NR	Pros	128	N vs. V
Piazza	2010	Italy	Dysplasia (mild-moderate), CIS, Cancer	61.79 (35–86)	96	58/38	Pros	96	N vs. V
Rahman	2012	India	Invasive carcinoma or all dysplasia	43 (26–60)	86	68/18	Pros	86	T vs. cy
Rajmohan	2012	India	Invasive carcinoma or all dysplasia	NR	30	NR	Pros	30	C vs. T
Ram	2005	Malaysia	Invasive carcinoma or all dysplasia	35–80 (56.75)	31	NR	Pros	31	C vs. T
Rana	2012	Germany	Invasive carcinoma or all dysplasia	62.5 ± 10.81	123	46/77	CS	123	A vs. V
Roblyer	2010	USA	Invasive carcinoma or all dysplasia	NR	72	NR	Pros	175	C vs. V
Sharma	2011	India	Invasive carcinoma or all dysplasia	44.34 ± 10.78	50	35/15	Pros	50	C vs. TS vs. cy
Shukla	2018	India	Invasive carcinoma or all dysplasia	21–60	42	37/5	Pros	42	C vs. T
Simonato	2017	Brazil	Invasive carcinoma or all dysplasia	52.13	15	11/4	Pros	15	A vs. V
Simonato	2019	Brazil	Invasive carcinoma or all dysplasia	NR	NR	NR	Pros	61	A vs. V
Ujaoney	2012	India	Invasive carcinoma or all dysplasia	44.4 (15)	55	51/4	Pros	99	C vs. T
Vashisht	2014	India	Invasive carcinoma or all dysplasia	NR	35	NR	Pros	35	C vs. T

Pros, Prospective; Retro, Retrospective; CS, Cross-sectional NR, Not reported; V, Visual examination; A, Autofluorescence; C, chemiluminescence; cy, cytology; N, Narrow band imaging; T, Toluidine blue; TP, Ture positive; FP, False positive; FN, False Negative; TN, Ture negative.

### Traditional meta-analysis

Table 2 presents the traditional meta-analysis of different adjunctive diagnostic tools on the sensitivity, specificity, negative predictive value, positive predictive value, and accuracy.

**Table 2:** Traditional meta-analysis of different diagnostic tools regarding sensitivity, specificity, negative predictive value, positive predictive value, and accuracy.

Diagnostic methods	Sensitivity	Specificity	Negative predictive values	Positive predictive value	Accuracy
Autofluorescence (9 studies)	0.8556 [0.7435; 0.9237	0.4882 [0.2871; 0.6933]	0.8655 [0.7219; 0.9411],	0.4933 [0.2699; 0.7194]	0.6638 [0.5143; 0.7864])
Chemiluminescence (11 studies)	0.8750 [0.7586; 0.9397]	0.5680 [0.2940; 0.8059]	0.8338 [0.6994; 0.9154]	0.6639 [0.3854; 0.8615]	0.7452 [0.5981; 0.8518]
Cytology (3 studies)	0.7212 [0.4517; 0.8904]	0.8623 [0.7269; 0.9364]	0.8491 [0.6478; 0.9451]]	0.7509 [0.5296; 0.8898]	0.8187 [0.6232; 0.9250]
Narrow band imaging (2 studies)	0.9035 [0.8341; 0.9458]	0.9480 [0.7571; 0.9907]	0.9350 [0.6152; 0.9923]	0.9364 [0.8725; 0.9694]	0.9422 [0.8062; 0.9846]
Toluidine blue (14 studies)	0.7142 [0.6069; 0.8018]	0.8115 [0.6792; 0.8974]	0.7078 [0.5299; 0.8389]	0.8114 [0.7099; 0.8832]	0.7585 [0.6641; 0.8330]
Visual examination (11 studies)	0.7608 [0.6253; 0.8584]	0.7952 [0.5930; 0.9119]	0.8014 [0.6378; 0.9024]	0.7671 [0.6140; 0.8722]	0.8030 [0.7360; 0.8563]

Autofluorescence results showed sensitivity: 0.8556 [0.7435; 0.9237], specificity: 0.4882 [0.2871; 0.6933], NPV: 0.8655 [0.7219; 0.9411], PPV: 0.4933 [0.2699; 0.7194]; and accuracy: 0.6638 [0.5143; 0.7864]). Overall, autofluorescence had high sensitivity and NPV poor specificity, PPV, and accuracy. Chemiluminescence showed sensitivity: 0.8750 [0.7586; 0.9397]; specificity: 0.5680 [0.2940; 0.8059]; NPV: 0.8338 [0.6994; 0.9154]; PPV: 0.6639 [0.3854; 0.8615]; and accuracy: 0.7452 [0.5981; 0.8518]]. Briefly, chemiluminescence showed similar adjunctive diagnostic power as autofluorescence. Toluidine blue results showed sensitivity: 0.7142 [0.6069; 0.8018]; specificity: 0.8115 [0.6792; 0.8974]; NPV: 0.7078 [0.5299; 0.8389]; PPV: 0.8114 [0.7099; 0.8832]; and accuracy: 0.7585 [0.6641; 0.8330]. Visual examination showed sensitivity: 0.7608 [0.6253; 0.8584]; specificity: 0.7952 [0.5930; 0.9119]; NPV: 0.8014 [0.6378; 0.9024]; PPV: 0.7671 [0.6140; 0.8722]; and accuracy: 0.8030 [0.7360; 0.8563]. Therefore, toluidine blue and visual examination showed similarly moderate adjunctive diagnostic power.

Cytology results showed sensitivity: 0.7212 [0.4517; 0.8904]; specificity: 0.8623 [0.7269; 0.9364]; NPV: 0.8491 [0.6478; 0.9451]; PPV: 0.7509 [0.5296; 0.8898]; and accuracy: 0.8187 [0.6232; 0.9250]. Therefore, cytology had high specificity and NPV. NBI showed sensitivity: 0.9035 [0.8341; 0.9458]; specificity: 0.9480 [0.7571; 0.9907]; NPV: 0.9350 [0.6152; 0.9923]; PPV: 0.9364 [0.8725; 0.9694]; and accuracy: 0.9422 [0.8062; 0.9846]. As a result, NBI had high values in all adjunctive diagnostic parameters. However, these two methods included few studies (two to three) in terms of adjunctive diagnostic accuracy, suggesting that our results should be interpreted with caution and further studies with more patients are required.

### Network meta-analysis

**Figure 2:**
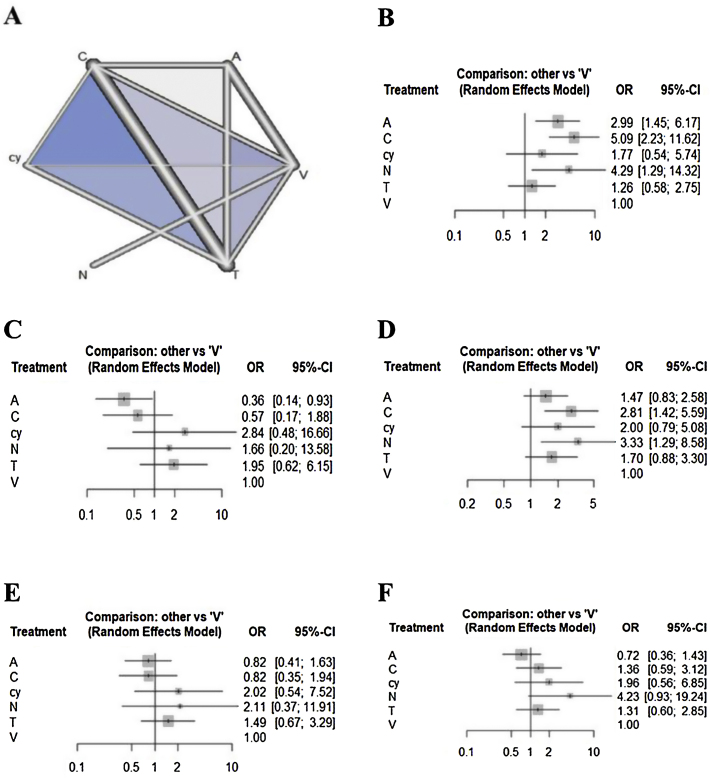
Evidence structure of eligible comparisons (A) and forest plots (B–F) for the network meta-analysis. Lines indicate direct comparisons in the eligible studies (A). The odds ratio of diagnostic accuracy is shown (B–F).

Regarding the outcome of sensitivity, chemiluminescence had the highest sensitivity compared with the visual examination (5.08 [95% CI 2.22 to 11.62]), followed by NBI (4.29 [95% CI 1.28 to 14.31]) and autofluorescence (2.98 [95% CI 1.44 to 6.17]). There were no significant differences between cytology, toluidine blue, and visual examination (Fig. 2). In terms of specificity, autofluorescence had the lowest specificity compared with visual examination (0.36 [95% CI 0.14 to 0.93]). Although chemiluminescence tended to be less specific compared to visual examination (0.57 [95% CI 0.17 to 1.88]), there were no significant differences in specificity among the other methods, including chemiluminescence. In terms of NPV, although all methods tended to have higher negative predictive value compared to visual examination, only chemiluminescence (2.81 [95% CI 1.41 to 5.59)]) and NBI (3.32 [95% CI 1.28 to 8.58]) showed a significant difference. In terms of PPV, although autofluorescence (0.81 [95% CI 0.41 to 1.63)]) and chemiluminescence (0.82 [95% CI 0.34 to 1.93)]) tended to have lower positive predictive value versus visual examination, there were no significant differences in specificity among all methods. In terms of accuracy, autofluorescence (0.7179 [95% CI 0.3597to 1.4327)]) tended to be less accurate and cytology (1.9591 [95% CI: 0.5604 to 6.8491)]) and NBI (4.2276 [95% CI 0.9287 to 19.2444)]) tended to be more accurate compared to visual examination. However, there were no significant differences in accuracy among all methods.

An evaluation of whether the direct and indirect comparisons were sufficiently similar in the NMA showed no global inconsistencies regarding sensitivity (*p* = 0.3878), specificity (*p* = 0.2333), NPV (*p* = 0.9884), PPV (*p* = 0.4254), or accuracy (*p* = 0.7371). Additionally, there were no local inconsistencies among outcomes (supplementary Tables 4 to 8).

### Results of the ranking hierarchy

The SUCRA values of the six adjunctive diagnostic methods were summarized and shown in Table 3. Considering the overall adjunctive diagnostic parameters, autofluorescence showed lower SUCRA values (sensitivity: 63.95%; specificity: 6.83%; NPV: 31.89%; PPV: 22.20%; accuracy: 8.01%). NBI showed higher SUCRA values (sensitivity: 77.91%; specificity: 63.78%; NPV: 83.54%; PPV: 72.52%; accuracy: 90.82%).

**Table 3:** Ranked probabilities of the effectiveness of different diagnostic tools on sensitivity, specificity, negative predictive value, positive predictive value, and accuracy.

Treatment	Sensitivity		Specificity		NPV	
	SUCRA	Rank	SUCRA	Rank	SUCRA	Rank
Visual examination	0.0920	6	0.4745	4	0.0460	6
Autofluorescence	0.6395	3	0.0683	6	0.3189	5
Chemiluminescence	0.8923	1	0.2382	5	0.8264	2
Cytology	0.3893	4	0.8346	1	0.5534	3
Narrow band imaging	0.7791	2	0.6378	3	0.8354	1
Toluidine blue	0.2078	5	0.7465	2	0.4200	4

Treatment	PPV		Accuracy	
	SUCRA	Rank	SUCRA	Rank
Visual examination	0.3800	4	0.2974	5
Autofluorescence	0.2220	6	0.0801	6
Chemiluminescence	0.2223	5	0.5220	3
Cytology	0.7752	1	0.7017	2
Narrow band imaging	0.7252	2	0.9082	1
Toluidine blue	0.6753	3	0.4906	4

SUCRA, surface under the cumulative ranking curve; PPV, positive predictive value.

### Detection of publication bias

**Figure 3:**
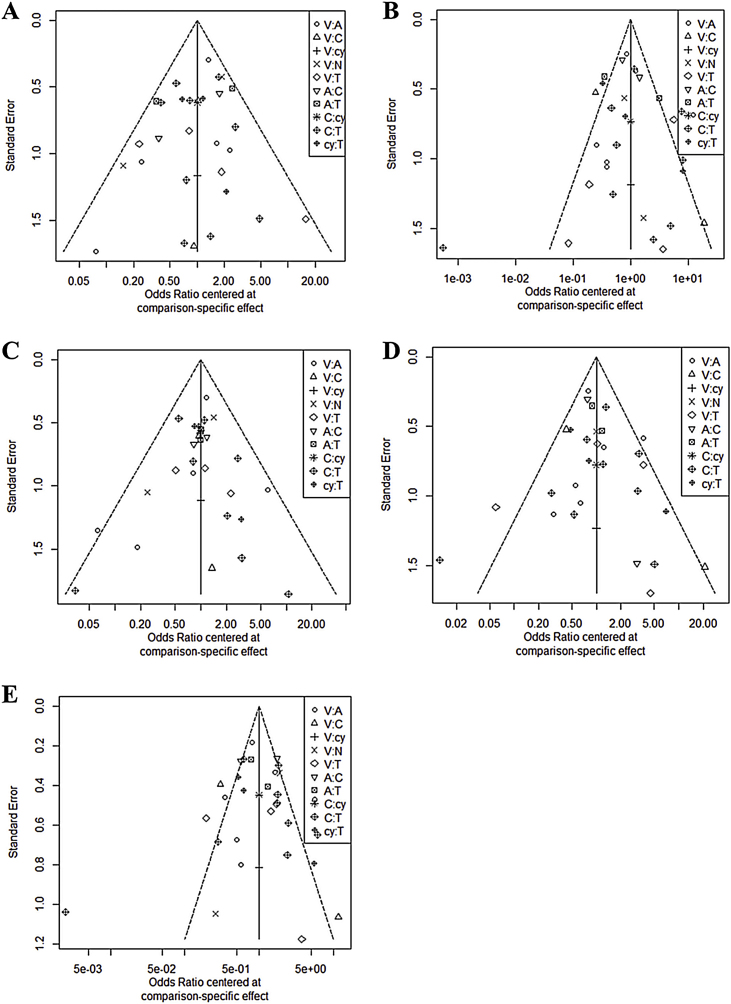
Funnel plot for publication bias. A, Sensitivity; B, Specificity; C, Negative predictive value; D, Positive predictive value; E, Accuracy.

In all six comparison-adjusted funnel plots (Fig. 3), scatters of the same symbols were visually symmetrical, which meant that publication bias was relatively low for sensitivity, specificity, negative predictive value, positive predictive value, and accuracy. Linear regression test of funnel plot asymmetry also showed no significant publication bias (*p* > 0.05).

## Discussion

Conventional visual assessment and tactile examination of the oral cavity remains the gold standard for the identification of oral mucosal lesions.^15^ However, an adjunctive method to detect OPMD has been reported showing a proper diagnostic yield.^25^ Therefore, our network conducted meta-analysis of those new studies, and included detailed comparisons of other adjunctive diagnostic tools with conventional visual examination. Previous meta-analyses or systemic reviews have documented the efficacy of several adjunctive diagnostic tools in detecting OPMD or cancerous lesions early and improving the survival rate for oral cancer.^40^ However, the limitations of these studies hindered their clinical relevance, as they consisted of separate diagnostic meta-analysis or descriptive reviews without statistical analysis; they could not assess multiple treatments or provide a ranking of their effectiveness.^35^ This makes it difficult for clinicians to select the optimal method among several methods demonstrated to be effective in previous meta-analyses or systemic reviews.

NMA is a novel analytic approach that enables simultaneous comparisons of multiple interventions. It also allows quantitative comparisons of treatments that previously had not been compared directly using direct and indirect data and combinations of evidence from different dimensions.^5^ This study used NMA to resolve the limitations of previous studies of adjunctive diagnostic methods for oral pre- or cancerous lesions that examined single, rather than multiple, treatment options or describe the systemic reviews without the presentation of objective values. Specifically, we conducted a systematic review using NMAs to rank the effects of five adjunctive diagnostic methods and visual examination used to detect the OPMD.

Regarding the five adjunctive diagnostic methods, in the autofluorescence method, the tissue autofluorescence produced by submucosal healthy tissues can be detected by fluorescence spectroscopy.^21^ Loss of autofluorescence occurs in OPMD due to biochemical changes of epithelial cells, inflammatory process, and angiogenesis occurred from the early neoplastic process.^41^ The optimum excited violet light of 400 nm has been verified to stimulate the oral mucous membrane which can be observed as “apple-green” light through optical filters. Lesion would be observed in well-demarcated dark areas due to the loss of tissue autofluorescence.^41^ The chemiluminescence method uses specific wavelengths that are absorbed by normal cells and reflected off abnormal cells due to their nuclear cytoplasmic ratio. Therefore, atypical mucosal abnormalities appear bright white.^25^ The NBI evaluates tissue characteristics using narrow-bandwidth filters that share absorption peaks with hemoglobin. This method uses blue and green light to reveal the blood vessels of the superficial mucous membrane and submucosa, enabling the detection of superficial and abnormal mucosal lesions.^42^ Toluidine blue is the most commonly used and widely studied vital staining technique. It is a dye with high affinity for acidic components and stains tissues with rich nucleic acids. Dysplastic or neoplastic cells contain wider intracellular canals compared to normal cells facilitating penetration of the dye. Oral exfoliative cytology has been used since the 1950s to collect epithelial cells for morphologic evaluation under the light microscope. With exfoliative cell collection using a bristle brush (brush cytology), full thickness epithelium including basal epithelial cells can be obtained. It is known to be a well-tolerated method that reduces the need for unnecessary surgical biopsies in clinically benign lesions.^17^

Autofluorescence and chemiluminescence showed higher sensitivity but lower specificity. These results indicate that there would be no difference between these light-based detection methods and clinical examination when evaluating obvious neoplastic lesions; however, when clinical examination yields negative neoplastic findings, these methods are more sensitive for identifying suspicious lesions. The high sensitivity of these methods can be attributed to the increased brightness and clarity of oral lesions, which could support the idea that these devices can detect new lesions and reveal tumor margins.^15^ Thus, these devices would be more effective than clinical examination in identifying non-symptomatic and clinically obscure lesions.^15^, ^26^

However, the pooled specificities were lower compared with those for clinical examination, mostly due to the false-positive results obtained with these devices. Thus, these may be useful for identifying all lesion types, but might be not reliable for distinguishing benign oral lesions from dysplasia or squamous cell carcinoma.^43^ The poor specificity could be explained by the mechanism of autofluorescence and chemiluminescence.^19^, ^44^ For chemiluminescent mixtures, we included an acetic acid pre-rinse to remove debris and glycoprotein layers, thereby increasing penetration and light reflection. However, acetic acid causes cell dehydration and protein coagulation, which reduces epithelial transparency. This reduced transparency could cause the aceto-white appearance of white lesions.^44^ Additionally, the use of an acetic acid pre-rinse could stimulate salivary gland secretion. This result in significant mucosal surface reflectivity could make it difficult to identify lesion boundaries.^11^

The high false-positive rate raised concerns of its potential risks, such as causing unnecessary stress and fear in patients, as well as increased morbidity due to surgical procedures for unnecessary biopsies.^19^ In addition, chemiluminescence screening has several limitations, such as the necessity of a dark environment, high cost, absence of a permanent record (except for photographs), and inability to measure visualization results objectively.^45^ The results of the present study also suggest that these methods could not provide a substitute for clinical examination of malignant and potentially malignant lesions in the oral mucosa.

The diagnostic accuracy of toluidine blue and oral exfoliative cytology tended to be higher than clinical examination, but there were no significant differences. In addition, the Material Data Safety Sheet indicates that toluidine blue could have hazardous effects if swallowed.^46^ Toluidine blue is not only expensive but also has relatively toxic effects on fibroblasts, inducing mutagenesis of stained cells under high energy irradiation.^47^ Oral exfoliative cytology also adds to the cost and delays the definite diagnosis.^48^ Therefore, the results of this meta-analysis indicate that these methods would not have additional advantages over their disadvantages and, thus, not constitute a convenient replacement for conventional screening tests that use standard overhead light.

In our study, NBI was in the top 3 in all diagnostic accuracy parameters. NBI would improve diagnostic sensitivity of a conventional endoscopic examination to assess tissue characteristics using narrow bandwidth filters with absorption peaks in hemoglobin. The NBI uses two light spectrums (blue and green), which can display blood vessels in superficial mucosa and submucosal areas. NBI could differentiate superficial mucosal lesions not detected under standard white light imaging endoscopy making it a useful tool for precise pathologic diagnosis and early diagnosis of oral premalignant or cancerous lesions.

There were some limitations to this study. There may be significant limitations for generalization due to the very small numbers of enrolled studies (from two studies). Second, although there have been two methods including mucosal patterns (well-demarcated brownish areas)^24^, ^49^ and vascular pattern (IPCL classification)^49^, ^50^ for discriminating pathologic lesions from benign mucosal lesions, only mucosal patterns (well-demarcated brownish areas) were included to compare the utility of NBI for aiding the detection of OPMD and malignant lesions in the oral cavity. In the view of these limitations, we need to include more reports with standardized assessment method in future studies to support or generalize our positive results for NBI. Third, since this article has judged based on one criterion (diagnostic accuracy), the clinically important values each of five adjunctive diagnostic tools has can be neglected.

Our results suggest that the current non-invasive adjunctive methods (except NBI) to detect OPMD might not improve the diagnostic accuracy compared to conventional examination. However, autofluorescence, chemiluminescence, and NBI are useful for mass screening and enable more accurate real-time optical diagnoses. Toluidine blue could make the biopsy site more clear for ambiguous lesions. Cytology could help determine whether real biopsy is necessary in patients with contraindications to surgical procedures, is useful for performing cell type evaluation on the oral epithelium itself and could be useful when it is difficult to construct a population screening setting. Therefore, in determining whether to use these adjunctive diagnostic tools, clinical situations should be carefully considered. Although NBI would be a good tool for adjunctive diagnosis of OPMD, more studies with standardized diagnostic criteria are required to support the usefulness of NBI.

## Conclusion

The results of our NMA showed that only NBI would be useful for detecting OPMD. The other methods (autofluorescence, chemiluminescence, cytology, and toluidine blue) have little benefit compared to conventional examination. In view of their cost and adverse effects, these methods would be not recommended as adjunctive diagnostic tools. For the utilization of NBI, further studies with standardized assessment method are required to support our results.

## Funding

This research was supported by the Basic Science Research Program through the 10.13039/501100003725National Research Foundation of Korea (NRF) funded by the 10.13039/501100003766Ministry of Education (2020R1I1A1A01051844, 2018R1D1A1B07045421), the Bio & Medical Technology Development Program of the National Research Foundation (NRF) funded by the 10.13039/501100004083Ministry of Science & ICT (2018M3A9E8020856, 2019M3A9H2032424, 2019M3E5D5064110), and the Korea Health Industry Development Institute funded by the Ministry of Health and Welfare (HI14C3228). The sponsors had no role in the study design, data collection and analysis, decision to publish, or preparation of the manuscript.

## Conflicts of interest

The authors declare no conflicts of interest.

## References

[1] Zhang L., Williams M., Poh C.F., Laronde D., Epstein J.B., Durham S. (2005). Toluidine blue staining identifies high-risk primary oral premalignant lesions with poor outcome. Cancer Res.

[2] Rahman F., Tippu S.R., Khandelwal S., Girish K.L., Manjunath B.C., Bhargava A. (2012). A study to evaluate the efficacy of toluidine blue and cytology in detecting oral cancer and dysplastic lesions. Quintessence Int.

[3] Awan K., Yang Y., Morgan P., Warnakulasuriya S. (2012). Utility of toluidine blue as a diagnostic adjunct in the detection of potentially malignant disorders of the oral cavity – a clinical and histological assessment. Oral Dis.

[4] Thomson P.J. (2002). Field change and oral cancer: new evidence for widespread carcinogenesis?. Int J Oral Maxillofac Surg.

[5] Rouse B., Chaimani A., Li T. (2017). Network meta-analysis: an introduction for clinicians. Intern Emerg Med.

[6] Ali Channa S., Surwaich A., Tariq U., Iqbal W. (2019). Methylene blue and lugolâ’s iodine as an adjunctive tool for early diagnosis of premalignant oral lesions. J Rawalpindi Med Col (JRMC).

[7] Simonato L.E., Tomo S., Miyahara G.I., Navarro R.S., Villaverde A. (2017). Fluorescence visualization efficacy for detecting oral lesions more prone to be dysplastic and potentially malignant disorders: a pilot study. Photodiagnosis Photodyn Ther.

[8] Simonato L.E., Tomo S., Scarparo Navarro R., Balbin Villaverde A.G.J. (2019). Fluorescence visualization improves the detection of oral, potentially malignant, disorders in population screening. Photodiagnosis Photodyn Ther.

[9] Allegra E., Lombardo N., Puzzo L., Garozzo A. (2009). The usefulness of toluidine staining as a diagnostic tool for precancerous and cancerous oropharyngeal and oral cavity lesions. Acta Otorhinolaryngol Ital.

[10] Amirchaghmaghi M., Mohtasham N., Delavarian Z., Shakeri M.T., Hatami M., Mosannen Mozafari P. (2018). The diagnostic value of the native fluorescence visualization device for early detection of premalignant/malignant lesions of the oral cavity. Photodiagnosis Photodyn Ther.

[11] Awan K.H., Morgan P.R., Warnakulasuriya S. (2015). Assessing the accuracy of autofluorescence, chemiluminescence and toluidine blue as diagnostic tools for oral potentially malignant disorders – a clinicopathological evaluation. Clin Oral Investig.

[12] Bhatia N., Matias M.A., Farah C.S. (2014). Assessment of a decision-making protocol to improve the efficacy of VELscope™ in general dental practice: a prospective evaluation. Oral Oncol.

[13] Chaudhari A., Hegde-Shetiya S., Shirahatti R., Agrawal D. (2013). Comparison of different screening methods in estimating the prevalence of precancer and cancer amongst male inmates of a jail in Maharashtra, India. Asian Pac J Cancer Prev.

[14] Chaudhary A., Manjunatha M., Gupta I. (2013). Evaluation of efficacy of toluidine blue in the detection of potentially malignant disorders. J Adv Med Dent Sci.

[15] Epstein J.B., Silverman S., Epstein J.D., Lonky S.A., Bride M.A. (2008). Analysis of oral lesion biopsies identified and evaluated by visual examination, chemiluminescence and toluidine blue. Oral Oncol.

[16] Farah C.S., McIntosh L., Georgiou A., McCullough M.J. (2012). Efficacy of tissue autofluorescence imaging (VELScope) in the visualization of oral mucosal lesions. Head Neck.

[17] Güneri P., Epstein J.B., Kaya A., Veral A., Kazandı A., Boyacioglu H. (2011). The utility of toluidine blue staining and brush cytology as adjuncts in clinical examination of suspicious oral mucosal lesions. Int J Oral Maxillofac Surg.

[18] Hanken H., Kraatz J., Smeets R., Heiland M., Assaf A.T., Blessmann M. (2013). The detection of oral pre- malignant lesions with an autofluorescence based imaging system (VELscope) – a single blinded clinical evaluation. Head Face Med.

[19] Jayaprakash V., Sullivan M., Merzianu M., Rigual N.R., Loree T.R., Popat S.R. (2009). Autofluorescence-guided surveillance for oral cancer. Cancer Prev Res (Phila).

[20] Kammerer P.W., Rahimi-Nedjat R.K., Ziebart T., Bemsch A., Walter C., Al-Nawas B. (2015). A chemiluminescent light system in combination with toluidine blue to assess suspicious oral lesions-clinical evaluation and review of the literature. Clin Oral Investig.

[21] Mehrotra R., Singh M., Thomas S., Nair P., Pandya S., Nigam N.S. (2010). A cross-sectional study evaluating chemiluminescence and autofluorescence in the detection of clinically innocuous precancerous and cancerous oral lesions. J Am Dent Assoc.

[22] Mojsa I., Kaczmarzyk T., Zaleska M., Stypulkowska J., Zapala-Pospiech A., Sadecki D. (2012). Value of the ViziLite Plus System as a diagnostic aid in the early detection of oral cancer/premalignant epithelial lesions. J Craniofac Surg.

[23] Petruzzi M., Lucchese A., Nardi G.M., Lauritano D., Favia G., Serpico R. (2014). Evaluation of autofluorescence and toluidine blue in the differentiation of oral dysplastic and neoplastic lesions from non dysplastic and neoplastic lesions: a cross-sectional study. J Biomed Opt.

[24] Piazza C., Del Bon F., Paderno A., Grazioli P., Perotti P., Barbieri D. (2016). The diagnostic value of narrow band imaging in different oral and oropharyngeal subsites. Eur Arch Otorhinolaryngol.

[25] Rajmohan M., Rao U.K., Joshua E., Rajasekaran S.T., Kannan R. (2012). Assessment of oral mucosa in normal, precancer and cancer using chemiluminescent illumination, toluidine blue supravital staining and oral exfoliative cytology. J Oral Maxillofac Pathol.

[26] Ram S., Siar C.H. (2005). Chemiluminescence as a diagnostic aid in the detection of oral cancer and potentially malignant epithelial lesions. Int J Oral Maxillofac Surg.

[27] Rana M., Zapf A., Kuehle M., Gellrich N.C., Eckardt A.M. (2012). Clinical evaluation of an autofluorescence diagnostic device for oral cancer detection: a prospective randomized diagnostic study. Eur J Cancer Prev.

[28] Roblyer D., Kurachi C., Stepanek V., Schwarz R.A., Williams M.D., El-Naggar A.K. (2010). Comparison of multispectral wide-field optical imaging modalities to maximize image contrast for objective discrimination of oral neoplasia. J Biomed Opt.

[29] Sharma S., Mubeen (2011). Non-invasive diagnostic tools in early detection of oral epithelial dysplasia. J Clin Experimental Dent.

[30] Shukla A., Singh N.N., Adsul S., Kumar S., Shukla D., Sood A. (2018). Comparative efficacy of chemiluminescence and toluidine blue in the detection of potentially malignant and malignant disorders of the oral cavity. J Oral Maxillofac Pathol.

[31] Vashisht N., Ravikiran A., Samatha Y., Rao P.C., Naik R., Vashisht D. (2014). Chemiluminescence and toluidine blue as diagnostic tools for detecting early stages of oral cancer: an invivo Study. J Clin Diagn Res.

[32] Vu A.N., Matias M., Farah C.S. (2015). Diagnostic accuracy of narrow band imaging for the detection of oral potentially malignant disorders. Oral Dis.

[33] Yang S.W., Lee Y.S., Chang L.C., Chien H.P., Chen T.A. (2012). Clinical appraisal of endoscopy with narrow-band imaging system in the evaluation and management of homogeneous oral leukoplakia. ORL J Otorhinolaryngol Relat Spec.

[34] Baratloo A., Hosseini M., Negida A., El Ashal G. (2015). Part 1: simple definition and calculation of accuracy, sensitivity and specificity. Emerg (Tehran).

[35] Shim S., Yoon B.H., Shin I.S., Bae J.M. (2017). Network meta-analysis: application and practice using Stata. Epidemiol Health.

[36] Salanti G., Ades A.E., Ioannidis J.P. (2011). Graphical methods and numerical summaries for presenting results from multiple-treatment meta-analysis: an overview and tutorial. J Clin Epidemiol.

[37] Flather M.D., Farkouh M.E., Pogue J.M., Yusuf S. (1997). Strengths, and limitations of meta-analysis: larger studies may be more reliable. Control Clin Trials.

[38] van Valkenhoef G., Dias S., Ades A.E., Welton N.J. (2016). Automated generation of node-splitting models for assessment of inconsistency in network meta-analysis. Res Synth Methods.

[39] Higgins J.P., Jackson D., Barrett J.K., Lu G., Ades A.E., White I.R. (2012). Consistency, and inconsistency in network meta-analysis: concepts and models for multi-arm studies. Res Synth Methods.

[40] Macey R., Walsh T., Brocklehurst P., Kerr A.R., Liu J.L., Lingen M.W. (2015). Diagnostic tests for oral cancer and potentially malignant disorders in patients presenting with clinically evident lesions. Cochrane Database Syst Rev.

[41] Tomo S., Miyahara G.I., Simonato L.E. (2019). History and future perspectives for the use of fluorescence visualization to detect oral squamous cell carcinoma and oral potentially malignant disorders. Photodiagnosis Photodyn Ther.

[42] Adham M., Musa Z., Lisnawati Suryati I. (2017). Sensitivity and specificity of narrow-band imaging nasoendoscopy compared to histopathology results in patients with suspected nasopharyngeal carcinoma. J Phys Conf Ser.

[43] Paderni C., Compilato D., Carinci F., Nardi G., Rodolico V., Lo Muzio L. (2011). Direct visualization of oral-cavity tissue fluorescence as novel aid for early oral cancer diagnosis and potentially malignant disorders monitoring. Int J Immunopathol Pharmacol.

[44] Nagi R., Reddy-Kantharaj Y.B., Rakesh N., Janardhan-Reddy S., Sahu S. (2016). Efficacy of light-based detection systems for early detection of oral cancer and oral potentially malignant disorders: systematic review. Med Oral Patol Oral Cir Bucal.

[45] Farah C.S., McCullough M.J. (2007). A pilot case control study on the efficacy of acetic acid wash and chemiluminescent illumination (ViziLite) in the visualisation of oral mucosal white lesions. Oral Oncol.

[46] Soman C., Lingappa A., Mujib A. (2016). Topical methylene blue in-vivo staining as a predictive diagnostic and screening tool for oral dysplastic changes – a randomised case control study. Res Rev J Dental Sci.

[47] Nethan S., Raju S., Chandra S., Sah K. (2018). Diagnostic efficacy of 1% methylene blue vital staining in individuals suspected with oral potentially malignant disorders. Acta Scientific Dental Sciences.

[48] Kazanowska K., Hałoń A., Radwan-Oczko M. (2014). The role and application of exfoliative cytology in the diagnosis of oral mucosa pathology – contemporary knowledge with review of the literature. Adv Clin Exp Med.

[49] Yang S.W., Lee Y.S., Chang L.C., Hwang C.C., Luo C.M., Chen T.A. (2015). Clinical characteristics of narrow-band imaging of oral erythroplakia and its correlation with pathology. BMC Cancer.

[50] Yang S.W., Lee Y.S., Chang L.C., Hwang C.C., Chen T.A. (2014). Use of endoscopy with narrow-band imaging system in detecting squamous cell carcinoma in oral chronic non-healing ulcers. Clin Oral Investig.

