# Supplementary material for: Efficacy of non-invasive diagnostic methods in the diagnosis and screening of oral cancer and precancer
Source: Braz J Otorhinolaryngol. 2021 Feb 13;88(6):937–47. doi: 10.1016/j.bjorl.2020.12.019 (PMC9615541; doi:10.1016/j.bjorl.2020.12.019)
Supplement: Supplementary file 1 [file mmc1.doc]

**BJORL-D-20-00665 – Supplementary Material**

**Supplementary Table 1** Search strategy of pubmed.

| #1 | "Mouth Neoplasms"[MeSH Terms] OR "Mouth Neoplasm*"[Title/Abstract] OR "Mouth Tumor*"[Title/Abstract] OR "Mouth cancer"[Title/Abstract] OR "oral cancer"[Title/Abstract] |
| --- | --- |
| #2 | "Mouth"[MeSH Terms] OR mouth*[Title/Abstract] |
| #3 | Leukoplakia[MeSH Terms] OR Precancerous Conditions[MeSH Terms] OR "Carcinoma in Situ"[MeSH Terms] |
| #4 | Leukoplakia[Title/Abstract] OR "premalignant lesion*"[Title/Abstract] OR "Precancerous Condition*"[Title/Abstract] OR dysplasia[Title/Abstract] OR "Carcinoma in Situ"[Title/Abstract] |
| #5 | #2 AND (#3 OR #4) |
| #6 | **#1 OR #5** |
| #7 | "Optical Imaging"[MeSH Terms] OR "Luminescence"[MeSH Terms] OR "Cytological Techniques"[MeSH Terms] OR "Narrow Band Imaging"[MeSH Terms] OR "Tolonium Chloride"[MeSH Terms] |
| #8 | "Optical Imaging"[Title/Abstract] OR Luminescence[Title/Abstract] OR "Cytological Techniques"[Title/Abstract] OR "Narrow Band Imaging"[Title/Abstract] OR "Tolonium Chloride"[Title/Abstract] OR autofluorescence[Title/Abstract] OR chemiluminescence[Title/Abstract] OR "toluidine blue"[Title/Abstract] OR "fluorescence imaging"[Title/Abstract] |
| #9 | **#7 OR #8** |
| #10 | "Sensitivity and Specificity"[MeSH Terms] OR "diagnostic accuracy"[Title/Abstract] OR "sensitivity and specificity"[Title/Abstract] |
| #11 | **#6 AND #9 AND #10** |
| #12 | **#11 NOT (Case Reports [publication type] OR Review [publication type])** |

**Supplementary Table 2** Search strategy of Embase.

| #1 | Mouth tumor'/exp/mj |
| --- | --- |
| #2 | Mouth tumor*':ab,ti OR 'mouth neoplasm*':ab,ti OR 'mouth cancer':ab,ti OR 'oral cancer':ab,ti |
| #3 | **#1 OR #2** |
| #4 | Mouth'/exp OR 'mouth':ti,ab |
| #5 | Leukoplakia'/exp OR 'precancer and cancer-in-situ'/exp OR 'carcinoma in situ'/exp |
| #6 | Leukoplakia':ti,ab OR 'premalignant lesion':ti,ab OR 'Precancerous Condition*':ti,ab OR 'dysplasia':ti,ab OR 'carcinoma in situ':ti,ab |
| #7 | **#4 AND (#5 OR #6)** |
| #8 | **#3 OR #7** |
| #9 | Fluorescence imaging'/exp OR 'luminescence'/exp OR 'cytology'/exp OR 'narrow band imaging'/exp OR 'tolonium chloride'/exp |
| #10 | Fluorescence imaging':ti,ab OR 'optical imaging':ti,ab OR luminescence:ti,ab OR 'cytological techniques':ti,ab OR 'narrow band imaging':ti,ab OR 'tolonium chloride':ti,ab OR autofluorescence:ti,ab OR chemiluminescence:ti,ab OR 'toluidine blue':ti,ab |
| #11 | **#9 OR #10** |
| #12 | Diagnostic accuracy':ti,ab OR 'sensitivity and specificity':ti,ab |
| #13 | Diagnostic accuracy'/exp OR 'sensitivity and specificity'/exp |
| #14 | **#12 OR #13** |
| #15 | **#8 AND #11 AND #14** |
| #16 | **#8 AND #11 AND #14 AND ([article]/lim OR [article in press]/lim) AND [embase]/lim** |

**Supplementary Table 3** Search strategy of Cochrane data base.

| #1 | MeSH descriptor: [Mouth Neoplasms] explode all trees |
| --- | --- |
| #2 | (Mouth Neoplasm*):ti,ab,kw OR (Mouth Tumor*):ti,ab,kw OR (Mouth Cancer):ti,ab,kw OR (Oral Cancer):ti,ab,kw |
| #3 | **#1 OR #2** |
| #4 | MeSH descriptor: [Mouth] explode all trees |
| #5 | (mouth*):ti,ab,kw |
| #6 | #4 OR #5 |
| #7 | MeSH descriptor: [Leukoplakia] explode all trees |
| #8 | MeSH descriptor: [Precancerous Conditions] explode all trees |
| #9 | MeSH descriptor: [Carcinoma in Situ] explode all trees |
| #10 | #7 OR #8 OR #9 |
| #11 | (leukoplakia):ti,ab,kw OR (premalignant lesion*):ti,ab,kw OR (Precancerous Condition*):ti,ab,kw OR (dysplasia):ti,ab,kw OR (Carcinoma in Situ):ti,ab,kw |
| #12 | #6 AND (#10 OR #11) |
| #13 | **#3 OR #12** |
| #14 | MeSH descriptor: [Optical Imaging] explode all trees |
| #15 | MeSH descriptor: [Luminescence] explode all trees |
| #16 | MeSH descriptor: [Cytological Techniques] explode all trees |
| #17 | MeSH descriptor: [Narrow Band Imaging] explode all trees |
| #18 | MeSH descriptor: [Tolonium Chloride] explode all trees |
| #19 | #14 OR #15 OR #16 OR #17 OR #18 |
| #20 | (Optical Imaging):ti,ab,kw OR (Luminescence):ti,ab,kw OR (Cytological Techniques):ti,ab,kw OR (Narrow Band Imaging):ti,ab,kw OR (Tolonium Chloride):ti,ab,kw |
| #21 | (autofluorescence):ti,ab,kw OR (chemiluminescence):ti,ab,kw OR (toluidine blue):ti,ab,kw OR (fluorescence imaging):ti,ab,kw |
| #22 | **#19 OR #20 OR #21** |
| #23 | MeSH descriptor: [Sensitivity and Specificity] explode all trees |
| #24 | (diagnostic accuracy):ti,ab,kw OR (sensitivity and specificity):ti,ab,kw |
| #25 | **#23 OR #24** |
| **#26** | **#13 AND #22 AND #25** |

**Supplementary Table 4** Odds ratio with 95% Confidence Intervals and local inconsistencies from the network meta-analysis of sensitivity.

| **Visual examination** | 2.8777 [1.3180; 6.2831] | 3.043 [1.0278; 12.8944] | 9.3333 [0.9579; 90.9396] | 5.3428 [1.0444; 27.3310] | 0.8406 [0.1759; 4.0175] |
| --- | --- | --- | --- | --- | --- |
| 3.1292 [1.4160; 6.9155]  *p* = 0.4414 | **Autofluorescence** | 1.1893 [0.2713; 5.2137] | N/A | N/A | 0.6358 [0.0960; 4.2094] |
| 5.2101 [2.2133; 12.2648]  *p* = 0.3973 | 1.2158 [0.4147; 3.5645]  *p* = 0.4199 | **Chemiluminescence** | 2.494 [-0.3759; 0.5899] | N/A | 0.3128 [0.1396; 0.7008] |
| 1.8099 [0.5391; 6.0761]  *p* = 0.1551 | 0.5840 [0.1206; 2.8290]  N/A | 0.4804 [0.1221; 1.8902]  *p* = 0.8275 | cytology | N/A | 1.3162 [0.4624; 3.7461] |
| 4.3913 [1.2682; 15.2056]  N/A | 1.7487 [0.2562; 11.9346]  N/A | 1.4383 [0.2079; 9.9528]  N/A | 2.9941 [0.3243; 27.6431]  N/A | **Narrow band imaging** | N/A |
| 1.2889 [0.5758; 2.8854]  *p* = 0.5336 | 0.4211 [0.1485; 1.1944]  *p* = 0.4970 | 0.3464 [0.1675; 0.7162]  *p* = 0.5003 | 0.7210 [0.2008; 2.5885]  *p* = 0.1246 | 0.2408 [0.0357; 1.6267]  N/A | **Toluidine blue** |

Treatments are reported in alphabetical order. The results of direct comparisons are shown in the upper triangle, and the NMA results and local inconsistencies are shown in the lower triangle. For both, the estimates were obtained based on comparisons of the row-indicated treatments with the column-indicated treatments. N/A, Not Available.

**Supplementary Table 5** Odds ratio with 95% confidence intervals and local inconsistencies from the network meta-analysis of Specificity.

| **Visual examination** | 0.3475 [0.1592; 0.7587] | 0.9181 [0.0507; 16.6161] | 3.2400 [0.3160; 33.2218] | 1.4999 [0.9296; 2.4199] | 1.0226 [0.1152; 9.0783] |
| --- | --- | --- | --- | --- | --- |
| 0.2569 [0.0862; 0.7657]  *p* = 0.7458 | **Autofluorescence** | 3.6671 [1.8947; 7.0972] | N/A | N/A | 3.6209 [0.4262; 30.7633] |
| 0.4824 [0.1413; 1.6472]  *p* = 0.2929 | 2.4967 [0.7966; 7.8249]  *p* = 0.6068 | **Chemiluminescence** | 1.3068 [0.3100; 5.5093] | N/A | 4.6234 [1.4387; 14.8575] |
| 2.4722 [0.4093; 14.9336]  *p* = 0.7873 | 11.3698 [1.9156; 67.4836]  N/A | 4.5539 [0.9060; 22.8898]  *p* = 0.2613 | **Cytology** | N/A | 0.4009 [0.0824; 1.9508] |
| 1.6554 [0.1984; 13.8160]  N/A | 7.2333 [1.4153; 36.9679]  N/A | 2.8971 [0.4946; 16.9693]  N/A | 0.6362 [0.0699; 5.7924]  N/A | **Narrow band imaging** | N/A |
| 1.6703 [0.5119; 5.4500]  *p* = 0.4472 | 7.3616 [2.3542; 23.0192]  *p* = 0.3117 | 2.9485 [1.2254; 7.0946]  *p* = 0.0665 | 0.6475 [0.1463; 2.8654]  *p* = 0.0630 | 1.0177 [0.1752; 5.9121]  N/A | **Toluidine blue** |

Treatments are reported in alphabetical order. The results of direct comparisons are shown in the upper triangle, and the NMA results and local inconsistencies are shown in the lower triangle. For both, the estimates were obtained based on comparisons of the row-indicated treatments with the column-indicated treatments. N/A, Not Available.

**Supplementary Table 6** Odds ratio with 95% confidence intervals and local inconsistencies from the network meta-analysis of NPV.

| **Visual examination** | 1.3124 [0.7112; 2.4217] | 2.8650 [0.9381; 8.7503] | 6.4800 [0.7283; 57.6564] | 4.4604 [0.8252; 24.1079] | 1.0050 [0.3415; 2.9581] |
| --- | --- | --- | --- | --- | --- |
| 1.4282 [0.7659; 2.6634]]  *p* = 0.5425 | **Autofluorescence** | 1.6419 [0.6754; 3.9912] | N/A | N/A | 1.5852 [0.6979; 3.6007] |
| 2.7719 [1.3566; 5.6637]  *p* = 0.7423 | 2.0076 [1.0461; 3.8526]  *p* = 0.5959 | **Chemiluminescence** | 0.6098 [0.2030; 1.8325] | N/A | 0.6793 [0.3937; 1.1720] |
| 1.9776 [0.7532; 5.1924]  *p* = 0.8684 | 1.5388 [0.6085; 3.8914]  N/A | 0.7665 [0.3386; 1.7354]  *p* = 0.6345 | **Cytology** | N/A | 1.1185 [0.5551; 2.2538] |
| 3.3931 [1.2677; 9.0821]  N/A | 2.8158 [0.9722; 8.1552]  N/A | 1.4026 [0.4359; 4.5129]  N/A | 1.8298 [0.4813; 6.9564]  N/A | **Narrow band imaging** | **N/A** |
| 1.6648 [0.8356; 3.3171]  *p* = 0.4472 | 1.3238 [0.7062; 2.4817]  *p* = 0.5996 | 0.6594 [0.4081; 1.0657]  *p* = 0.6849 | 0.8603 [0.4072; 1.8174]  *p* = 0.9077 | 0.4702 [0.1481; 1.4928]  N/A | **Toluidine blue** |

Treatments are reported in alphabetical order. The results of direct comparisons are shown in the upper triangle, and the NMA results and local inconsistencies are shown in the lower triangle. For both, the estimates were obtained based on comparisons of the row-indicated treatments with the column-indicated treatments. N/A, Not Available.

**Supplementary Table 7** Odds ratio with 95% confidence intervals and local inconsistencies from the network meta-analysis of PPV.

| **Visual examination** | 0.5123 [0.2494; 1.0523] | 0.9871 [0.4158; 2.3436] | 4.6667 [0.4178; 52.1210] | 2.1070 [0.7394; 6.0043] | 1.6125 [0.5174; 5.0260] |
| --- | --- | --- | --- | --- | --- |
| 0.7622 [0.3464; 1.6772]  *p* = 0.4571 | **Autofluorescence** | 1.0597 [0.5904; 1.9021] | N/A | N/A | 1.5175 [0.8550; 2.6933] |
| 0.7854 [0.3184; 1.9375]  *p* = 0.5126 | 1.3341 [0.6038; 2.9475]  *p* = 0.4528 | **Chemiluminescence** | 0.8594 [0.1875; 3.9390] | N/A | 1.8753 [1.0553; 3.3326] |
| 1.9810 [0.5072; 7.7370]  *p* = 0.4857 | 3.2985 [0.8869; 12.2672]  N/A | 2.4725 [0.7479; 8.1744]  *p* = 0.2134 | **Cytology** | N/A | 1.9472 [0.5168; 7.3363] |
| 2.1070 [0.3467; 12.8055]  N/A | 3.5891 [0.5681; 22.6765]  N/A | 2.6904 [0.4164; 17.3837]  N/A | 1.0881 [0.1283; 9.2291]  N/A | **Narrow band imaging** | **N/A** |
| 1.4410 [0.6245; 3.3249]  *p* = 0.6683 | 2.4207 [1.1224; 5.2205]  *p* = 0.2446 | 1.8145 [1.0133; 3.2492]  *p* = 0.6704 | 0.7339 [0.2411; 2.2336]  *p* = 0.0894 | 0.6744 [0.1049; 4.3363]  N/A | **Toluidine blue** |

Treatments are reported in alphabetical order. The results of direct comparisons are shown in the upper triangle, and the NMA results and local inconsistencies are shown in the lower triangle. For both, the estimates were obtained based on comparisons of the row-indicated treatments with the column-indicated treatments. N/A, Not Available.

**Supplementary Table 8** Odds ratio with 95% confidence intervals and local inconsistencies from the network meta-analysis of Accuracy.

| **Visual examination** | 0.5984 [0.2622; 1.3658] | 0.5938 [0.1776; 1.9848] | 5.4265 [1.0976; 26.8291] | 4.7035 [0.7178; 30.8200] | 1.4448 [0.3929; 5.3134] |
| --- | --- | --- | --- | --- | --- |
| 0.6057 [0.2772; 1.3235]  *p* = 0.9948 | **Autofluorescence** | 2.2956 [0.7745; 6.8039] | N/A | N/A | 1.9651 [1.2290; 3.1421] |
| 1.2471 [0.5269; 2.9516]  *p* = 0.2224 | 2.0011 [0.7837; 5.1098]  *p* = 0.8219 | **Chemiluminescence** | 0.6711 [0.2784; 1.6177] | N/A | 1.5121 [0.6692; 3.4166] |
| 1.8407 [0.5128; 6.6070]  *p* = 0.3695 | 3.9130 [0.8773; 17.4544]  N/A | 1.9554 [0.5109; 7.4846]  *p* = 0.2530 | **cytology** | N/A | 0.5746 [0.1954; 1.6898] |
| 4.2633 [0.9148; 19.8695]  N/A | 9.0998 [1.3343; 62.0619]  N/A | 4.5474 [0.6456; 32.0319]  N/A | 2.3255 [0.2432; 22.2393]  N/A | **Narrow band imaging** | N/A |
| 1.2162 [0.5440; 2.7192]  *p* = 0.7340 | 2.6775 [1.0590; 6.7700]  *p* = 0.5396 | 1.3380 [0.6869; 2.6062]  *p* = 0.4352 | 0.6843 [0.1939; 2.4142]  *p* = 0.3254 | 0.2942 [0.0421; 2.0545]  N/A | **Toluidine blue** |

Treatments are reported in alphabetical order. The results of direct comparisons are shown in the upper triangle, and the NMA results and local inconsistencies are shown in the lower triangle. For both, the estimates were obtained based on comparisons of the row-indicated treatments with the column-indicated treatments. N/A, Not Available.
